# Encapsulating soluble active species into hollow crystalline porous capsules beyond integration of homogeneous and heterogeneous catalysis

**DOI:** 10.1093/nsr/nwz147

**Published:** 2019-10-01

**Authors:** Guorui Cai, Meili Ding, Qianye Wu, Hai-Long Jiang

**Affiliations:** Hefei National Laboratory for Physical Sciences at the Microscale, CAS Key Laboratory of Soft Matter Chemistry, Collaborative Innovation Center of Suzhou Nano Science and Technology, Department of Chemistry, University of Science and Technology of China, Hefei 230026, China

**Keywords:** metal–organic frameworks, hollow capsules, molecular catalyst, heterogeneous catalysis, cascade reaction

## Abstract

Homogeneous molecular catalysts and heterogeneous catalysts possess complementary strengths, and are of great importance in laboratory/commercial procedures. While various porous hosts, such as polymers, carbons, silica, metal oxides and zeolites, have been used in an attempt to heterogenize homogeneous catalysts, realizing the integration of both functions at the expense of discounting their respective advantages, it remains a significant challenge to truly combine their intrinsic strengths in a single catalyst without compromise. Here, we describe a general template-assisted approach to incorporating soluble molecular catalysts into the hollow porous capsule, which prevents their leaching due to the absence of large intergranular space. In the resultant yolk (soluble)–shell (crystalline) capsules, the soluble yolks can perform their intrinsic activity in a mimetic homogeneous environment, and the crystalline porous shells endow the former with selective permeability, substrate enrichment, size-selective and heterogeneous cascade catalysis, beyond the integration of the respective advantages of homogeneous and heterogeneous catalysts.

## INTRODUCTION

Molecular catalysts are of great importance in both laboratory and commercial procedures due to their high activity and selectivity toward many reactions. However, these homogeneous catalysts suffer from intrinsic difficulty in separation/reuse and the tendency toward degradation or aggregation [1–16]. Encapsulation of these homogeneous catalysts in porous solid materials might be an effective solution to address the above issues [1–16]. To this end, a prerequisite for the porous hosts is to have uniform pore openings that are smaller than the soluble catalysts but larger than substrates/products so as to guarantee the fast diffusion of the latter. While the use of porous inorganic solids such as zeolites has been attempted, their microporous structures are unfortunately limited to the encapsulation of small guest species and readily cause mass transfer resistance [8–10]. Moreover, their preparation usually requires special templates (such as hard/soft templates or structure-directing agents) to create pores and subsequent high-temperature calcination to remove the templates, which is unfavorable to the encapsulation of soluble active species [8–10]. In this respect, metal–organic frameworks (MOFs), a class of crystalline porous materials assembled by metal ions and organic linkers, are ideal candidates [17–26]. The high porosity of MOFs facilitates access to confined active species and rapid mass transport. Their well-defined and tunable pores would impart the size-selective catalysis. Given these merits, researchers have used some MOFs to directly incorporate molecular catalysts [13–16]. Unfortunately, compared with free molecular catalysts, simply introducing them into the narrow pores of MOFs usually leads to decreased activity and instability of the resultant catalysts, due to the pore blocking, active molecule distortion, and the leaching effect [13–16]. Moreover, the microporous feature of MOFs significantly limits the size and loading amount of active guests. Alternatively, while the assembly of active complexes onto the framework by pre-synthesis of particular ligands or postsynthetic approach is feasible, this usually requires complicated procedures to modify the molecular catalysts and/or MOFs with particular groups (for example, -NH_2_). This solution not only imposes a limit on the active complexes and MOFs, but also sometimes changes the intrinsic behavior of the molecular catalyst once fixed to the framework [11,12]. Therefore, the published methods usually give a compromised porosity of the heterogeneous catalysts and catalytic activity of homogeneous catalysts, and it is highly desirable yet challenging to combine the strengths of homogeneous guests and MOF hosts for enhanced and stable heterogeneous catalysis without compromise.

To completely integrate the potential strengths of these two types of materials, it would be prudent to fabricate hollow MOF capsules [27–32] for the accommodation of homogeneous active guests. The large interior cavity of a MOF capsule will behave as a nanoreactor and mimic a homogeneous environment, where the soluble guests will exert their intrinsic catalytic activity. The MOF shell with accurate pore opening sizes not only allows the diffusion/transport of substrates/products and endows size-selective catalysis, but also prevents guest leaching and guarantees recyclability [33–39]. Motivated by these advantages, several researchers have reported on the encapsulation of large functional species, such as metal nanoparticles, enzymes (normally >2 nm in size due to the large intergranular spaces in the shell), etc., into hollow MOF nanospheres to provide yolk–shell catalysts [33–39]. However, to our knowledge, thus far there have been no published studies on entrapping homogeneous molecular (or metal complexes, typically <2 nm) catalysts into hollow MOF capsules for catalysis. The main challenges might lie in the following: (i) the creation of a continuous and stable MOF shell without defect (i.e. large intergranular space) is indispensable for the perfect enveloping of soluble catalysts; (ii) the soluble guests should be encapsulated prior to the assembly of hollow MOF capsules; and (iii) the MOF shell should be thin and sufficiently permeable for mass transfer not to be disturbed [33–35].

**Figure 1.**
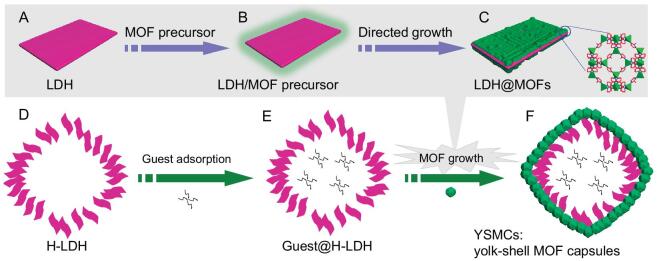
Schematic illustration showing the fabrication of yolk–shell MOF capsules. (A–C) Template-directed MOF growth on LDH. (D–F) Incorporation of soluble catalyst into H-LDH followed by MOF growth to produce the yolk–shell MOF capsules.

Here, we describe the development of a template-directed strategy for the controllable fabrication of hollow MOF capsules with a defect-free and stable shell (Fig. 1A–C). To demonstrate the generality of this approach, a variety of hollow MOF capsules ZIF-8 (also named MAF-4, Zn(mim)_2_, Hmin = 2-methylimidazole) [40,41], ZIF-67 (Co(mim)_2_) [42] and MOF-74 (also named CPO-27, Ni_2_(dhtp) or Cu_2_(dhtp), H_4_dhtp = 2,5-dihydroxyterephthalia acid) [43,44] have been successfully prepared (Supplementary Fig. 1 in the online supplementary material). The hollow templates constituted by layered double hydroxides (LDHs) feature large open channels between the LDH layers, and facilitate the adsorption/introduction of diverse homogeneous guests into the hollow space; they also drive the directed MOF growth on the LDH to eliminate the open channels (Fig. 1D–F). Therefore, such an approach is favorable to the entrapment of diverse molecular catalysts into the interior of hollow MOF structures. As a result, the homogeneous catalysts (yolks) have been encapsulated into hollow MOFs (shells), resulting in yolk–shell MOF capsules (denoted YSMCs), which not only exhibit excellent activity, selectivity and recyclability toward CO_2_ cycloaddition with epoxides under ambient conditions, but also achieve a one-pot cascade reaction based on the host–guest catalytic cooperation, far surpassing the corresponding single component counterparts, their physical mixture, and solid composite.

## RESULTS

### Synthesis and characterization of hollow H-LDH@ZIF-8 capsules

The LDHs with unsaturated metal sites featuring positively charged scaffolds are recognized as ideal templates for the directed growth of MOFs [45–47]. The positively charged scaffold induces the attachment of deprotonated ligands with negative charge onto its surface, which is followed by the coordination with metal ions and further MOF growth. The hollow LDH (H-LDH) nanostructure (Supplementary Figs 2 and 3), assembled from interlaced LDH nanoflakes, was prepared by stirring the ethanol solution of Co(NO_3_)_2_·6H_2_O and ZIF-67 at 90°C [45]. Then the H-LDH was employed as a template for epitaxial MOF growth to provide a hollow MOF capsule. The classical MOF, ZIF-8 [40,41], was first investigated as a representative. Typically, a methanol solution of 2-methyl-imidazole and Zn(NO_3_)_2_·6H_2_O was added to the H-LDH solution at room temperature for ZIF-8 assembly onto H-LDH to yield H-LDH@ZIF-8 with a hollow structure.

**Figure 2.**
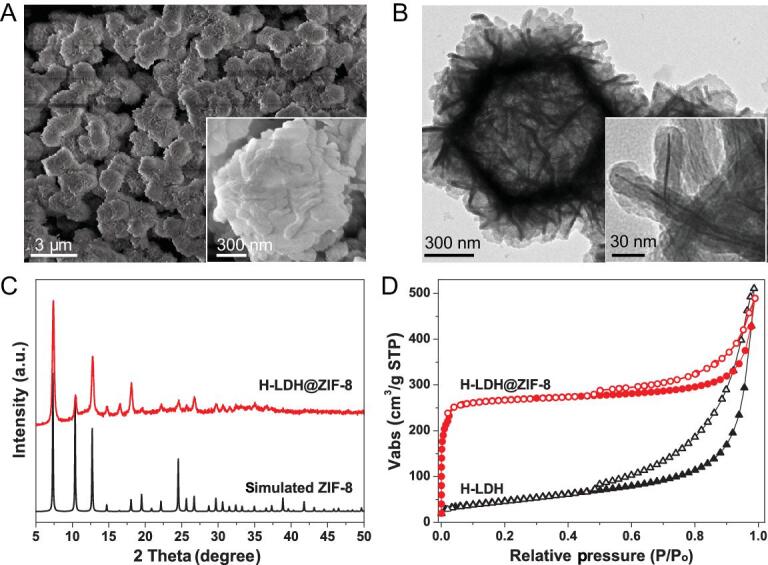
SEM and TEM images, powder XRD patterns, and N_2_ sorption isotherms. (A) SEM and (B) TEM images of H-LDH@ZIF-8 (inset: enlarged images). (C) Powder XRD patterns and (D) N_2_ sorption isotherms at 77 K.

Scanning electron microscopy (SEM) observation reveals the almost monodispersed particles of H-LDH@ZIF-8 in a polyhedral morphology with a sheet-like surface (Fig. 2A), basically inheriting the morphology of the original H-LDH template. In particular, the SEM images of broken capsules prove the presence of a hollow interior space in H-LDH@ZIF-8 (Supplementary Fig. 4). Transmission electron microscopy (TEM) images clearly display the hollow interior with void-free shells of ∼100 nm thickness assembled from nanoflakes (Fig. 2B). Close TEM observation of the nanoflake reveals a core–shell structure with a sharp contrast between the core and shell (Fig. 2B, inset). Energy-dispersive X-ray spectroscopy (EDS) analysis indicates a much higher Co signal in the center than in the shell where a higher Zn signal exists (Supplementary Fig. 5), revealing that part of the LDH template is left after ZIF-8 shell growth. Powder X-ray diffraction (XRD) confirms the ZIF-8 formation (Fig. 2C). N_2_ sorption of the H-LDH@ZIF-8 composite reveals a type-I isotherm at low pressures, a typical micropore characteristic similar to ZIF-8 (Fig. 2D and Supplementary Fig. 6). In addition, the type-IV curve with a pronounced hysteresis loop at high pressures demonstrates the presence of meso- and even macro-pores, attributable to the H-LDH (Supplementary Fig. 7). All the results unambiguously validate that ZIF-8 nanocrystals are grown onto H-LDH, resulting in the successful fabrication of H-LDH@ZIF-8.

### The formation mechanism of a hollow MOF capsule

To decode the formation mechanism of the template-directed MOF growth, the adding sequence of MOF precursors was altered for comparison. When the metal salts were added prior to the ligands, Zn^2+^ was repelled from the positively charged LDH skeleton. Accordingly, ZIF-8 nucleated separately rather than on the template, as demonstrated by SEM and TEM observation that only a part of ZIF-8 nanocrystals were located on the H-LDH (Supplementary Fig. 8). In contrast, the ligand added at first would be preferentially adsorbed onto H-LDH templates, driven by the abundant basic groups (i.e. OH^−^) on H-LDH templates to promote the deprotonation of imidazole ligand. The coordinatively unsaturated metal sites on the template, which act as growth sites, coordinate with the deprotonated ligands, facilitating the heterogeneous nucleation of a continuous ZIF-8 layer on the H-LDH surface (Fig. 2A and B). The results reveal that the adding sequence for the metal and ligand precursors plays a critical role in the growth of the MOF shell onto the H-LDH.

**Figure 3.**
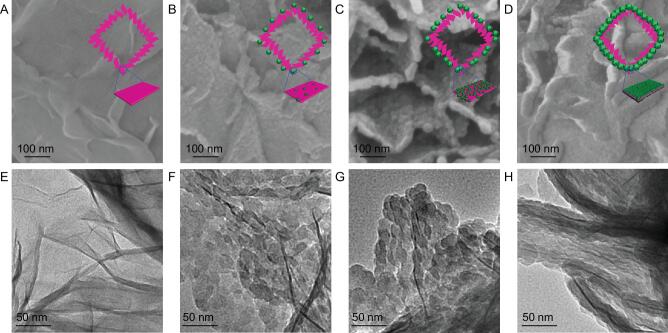
SEM and TEM images. (A–D) SEM and (E–H) TEM images showing the synthetic process of H-LDH@ZIF-8 by MOF growth at different reaction time lengths (0, 1, 3 and 5 min, respectively). Inset: schematic illustration of corresponding evolution process during the MOF growth.

The above growth process was further investigated by time-dependent experiments (Fig. 3). In the first 1 min, only a few isolated ZIF-8 nanoparticles with low contrast emerged on the LDH surface (Fig. 3B and F). Subsequently, those ZIF-8 nanoparticles grew and resulted in an almost continuous layer on the LDH (Fig. 3C and G). With prolonged reaction time, the ZIF-8 particles continued to grow and connect with each other to afford relatively uniform, continuous and compact MOF layers on both the top and bottom of the LDH nanoflakes, producing a sandwich-like LDH@MOF microstructure (Fig. 3D and H). The above observation clearly proves the efficacy of the MOF assembly on the LDH surface based on the template-directed growth mechanism, making the final hollow MOF capsule possible (Fig. 3, inset). In addition, the molecular guests can be easily pre-introduced into the H-LDH, and then sealed up by the MOF shell, yielding YSMCs.

### The generality of this template-directed strategy

To demonstrate the generality of this synthetic strategy, another two representative MOFs, ZIF-67 [42] and MOF-74 [43,44], were also employed to wrap onto H-LDH. The corresponding results, including SEM, TEM, XRD and N_2_ sorption, suggested that both MOFs were successfully grown on the H-LDH to produce H-LDH@ZIF-67 and H-LDH@MOF-74, respectively (Supplementary Figs 9 and 10).

### Capture and size-selective release of soluble molecules

To investigate the intactness of the resulting MOF shell, we adopted a hollow MOF capsule to capture soluble large-size molecules. The Coomassie

**Figure 4.**
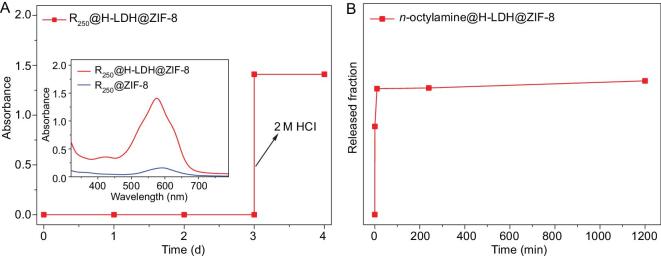
UV/Vis absorption spectra. UV/Vis absorption spectra recording the release of (A) R250 and (B) n-octylamine over different catalysts. Inset: R250 loading amount determined by UV/Vis spectroscopy upon acid etching.

Brilliant Blue R250 (R250) was employed as a visual probe for molecular uptake and release. The encapsulation of R250 into the cavity of H-LDH@ZIF-8 was performed to obtain R250@H-LDH@ZIF-8, which was then immersed in a methanol solution; the release of R250 from the capsules was monitored over time. Due to the molecular size of R250 being larger than the pore opening of ZIF-8, almost no R250 was detectable in the solvent using ultraviolet-visible spectroscopy (UV/Vis spectra)—even after 1 week (Fig. 4A). The results demonstrate that the MOF shell is defect-free and thus prevents the escape of molecular guests in the hollow space. Upon acid dissolution of the capsule, the R250 loading amount can be evaluated by UV/Vis spectra (Fig. 4A). To determine whether the dye molecules are located in the capsule interior or in the MOF intrinsic pores, the R250-loaded ZIF-8 (R250@ZIF-8) was also prepared by the in situ synthetic method for control. The absorbance intensity of R250@ZIF-8 is obviously lower than that of R250@H-LDH@ZIF-8 (Fig. 4A inset and Supplementary Fig. 11), suggesting that the dye should be primarily encased in the cavity of the MOF capsules and highlighting the importance of the hollow structure for hosting homogeneous guests. On the other hand, to examine the permeability of the capsule, a small molecule (n-octylamine) was encapsulated into H-LDH@ZIF-8 (denoted n-octylamine@H-LDH@ZIF-8). Extremely rapid release can be observed by gas chromatograph detection upon soaking the composite in methanol (Fig. 4B), implying unrestricted mass transport of the MOF shell for small molecules.

### Size-selective heterogeneous catalysis

**Figure 5.**
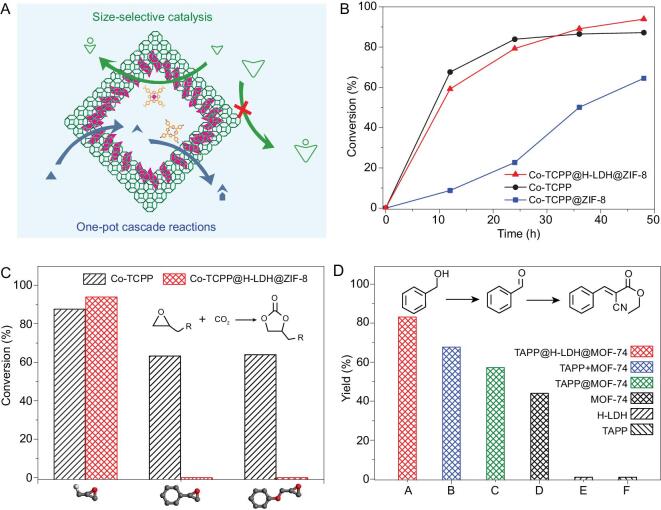
Size-selective and one-pot cascade catalysis. (A) Schematic illustration showing the YSMCs for size-selective CO_2_ cycloaddition and one-pot cascade oxidation/Knoevenagel condensation reactions. (B) Time conversion of the cycloaddition reaction between CO_2_ and epibromohydrin, (C) conversion of epoxides with different sizes, and (D) the yield of ethyl α-cyanocinnamate via the cascade reaction over different catalysts.

Given the encouraging results noted above, the hollow MOF capsule would be a promising nanoreactor for encapsulating catalytically active guests, which would guarantee their intrinsic activity, protect them from leaching, and even endow size-selective and cascade catalysis (Fig. 5A). Metalloporphyrin, such as Co-centered porphyrin (Co-TCPP), as an example of functional guests, can be readily encapsulated into H-LDH@ZIF-8 to afford yolk–shell Co-TCPP@H-LDH@ZIF-8 (Supplementary Figs 12 and 13). To demonstrate the advantages of the YSMCs, their catalytic performance in the cycloaddition of CO_2_ and epoxides to cyclic carbonates (Supplementary Fig. 14), an important reaction of upgrading CO_2_ to value-added chemicals [48–53], has been investigated. The Co-TCPP@H-LDH@ZIF-8 exhibits the highest conversion (94%) and selectivity (>99%), significantly higher activity than the solid composite of Co-TCPP in situ embedded in ZIF-8 (denoted Co-TCPP@ZIF-8, yield: 64%) and all other counterparts with a fixed catalyst amount, quantified by an inductively coupled plasma atomic emission spectrometer (ICP-AES), under very mild conditions (room temperature, 1 atm CO_2_) (Fig. 5B and Supplementary Fig. 15). The much-enhanced efficiency of the former should be attributed to the reduced diffusion limitation in the yolk–shell structure, offering the shortened distance of the reactants from the MOF shell to the active guests. In contrast, the bulk MOF system, where active guests are confined in the micropores of MOFs, performed a sluggish conversion especially at the early stage, caused by the considerable reduction in porosity due to the large foreign moieties occupying and even blocking the narrow apertures. Unexpectedly, the Co-TCPP@H-LDH@ZIF-8 possesses a superior activity to the free Co-TCPP (yield: 88%), which might be ascribed to the CO_2_ and substrate enrichment capability by the MOF shell, as indicated by adsorption experiments for CO_2_ and epoxide (Supplementary Figs 16 and 17). In addition, the void-free MOF shell is able to afford size-selective and recyclable catalysis, which cannot be achieved by the single Co-TCPP (Fig. 5C and Supplementary Figs 18 and 19). To examine the general application of the YSMCs, the small-size epoxides with various functional groups have been investigated (Supplementary Table 1). Nearly quantitative yields can be achieved in all reactions, reflecting the good substrate tolerance of the YSMCs toward the CO_2_ cycloaddition reaction.

To further manifest the superiority of the MOF capsule, a Mn-centered porphyrin has been encapsulated into a hollow H-LDH@ZIF-8 capsule, which also exhibits excellent activity and recyclability, surpassing the corresponding counterparts (Supplementary Figs 20 and 21).

In addition, the YSMC action of integrating active sites from host and guest might be employed for one-pot cascade reaction [54–58]. As a representative example, the amino functionalized porphyrin (TAPP) encapsulated in H-LDH@MOF-74 (denoted TAPP@H-LDH@MOF-74), where the MOF shell and the TAPP yolk behave as the active sites for the oxidation and Knoevenagel condensation of alcohols, respectively (Supplementary Fig. 22), presents excellent activity and recyclability, outperforming the corresponding single-component counterparts, their physical mixture and solid composite, in the one-pot tandem oxidation/Knoevenagel condensation of various alcohols to make α, β-unsaturated compounds (Fig. 5D, Supplementary Fig. 23 and Supplementary Table 2). The results have unambiguously demonstrated that the hollow MOF capsule incorporating active species presents superior synergistic properties, which successfully integrate the respective strengths of the MOF hosts and the guest molecular catalysts.

## DISCUSSION

In summary, we have presented a rational and versatile template-assisted strategy for the fabrication of various hollow MOF (i.e. ZIF-8, ZIF-67 and MOF-74) capsules with a continuous and thin MOF shell. The H-LDH template facilitates the encapsulation of diverse soluble active guests into the interior of the MOF capsule. Such a combination is able to perfectly integrate the homogeneous catalytic behavior of the guest with the heterogeneous and porous catalysis of MOF solids. As a result, such yolk (soluble)–shell (crystalline) capsules not only exert the high intrinsic activity of the homogeneous catalyst but also offer size-selective and recyclable catalysis, endowed by the void-free and crystalline MOF shell, as well as one-pot cascade catalysis by host–guest cooperation. As far as we know, this is the first report on the encapsulation of soluble species in hollow MOFs, in the absence of intergranular space, for catalysis. It is our belief that such a template-assisted strategy would open an avenue to the rational synthesis of diverse YSMCs for synergistically enhanced properties toward broad applications, especially in catalysis.

## METHODS

### Synthesis of H-LDH@ZIF-8

The ethanol solution of H-LDH (2 mL) was separated by centrifugation and washed with methanol several times. The resulting precipitate was then dispersed in methanol (2 mL); following this, the methanol solutions of 2-methylimidazole (45 mg/mL, 2 mL) and zinc nitrate hexahydrate (10 mg/mL, 2 mL) were added in sequence. After proceeding at room temperature for 2 h, the precipitate was recovered and washed with methanol several times. The as-prepared sample was then dried in a vacuum oven at 85°C for 12 h to yield H-LDH@ZIF-8.

### The uptake and release of large-sized molecules

In a typical experiment, 2 mL of an ethanol solution of H-LDH was isolated by centrifugation and washed with methanol several times. The resulting sample (dispersed in 1.5 mL of methanol) was soaked in a methanol solution (2 mg/mL, 0.5 mL) of R250. After stirring for 2 h at room temperature, a methanol solution (45 mg/mL, 2 mL) of 2-methylimidazole was added under gentle stirring, and then a methanol solution (10 mg/mL, 2 mL) of zinc nitrate hexahydrate was added. After proceeding at room temperature for 5 h, the precipitate was washed with methanol several times and then dried in a vacuum oven at 85°C for 12 h to yield R250@H-LDH@ZIF-8.

The procedures for R250@ZIF-8 followed the preparation of R250@H-LDH@ZIF-8 described above, except that the methanol solution of H-LDH was replaced by methanol only.

For the releasing experiment, the above samples (5 mg) were immersed in methanol (3 mL) for the required time. The supernatant was monitored periodically using a UV-Vis spectrophotometer. The total content of R250 within the samples was measured by the UV-Vis spectrophotometer upon dissolving R250@H-LDH@ZIF-8 and R250@ZIF-8 with 1 M HCl.

### The uptake and release of small-sized molecules

The synthetic procedure for n-octylamine@H-LDH@ZIF-8 was followed by the preparation of R250@H-LDH@ZIF-8 described above, except that the methanol solution of R250 was replaced by n-octylamine (0.5 mL). The reaction was allowed to proceed at room temperature for 5 h, and then the precipitate was carefully washed with methanol. For releasing, the above sample was directly soaked in methanol (1 mL) for the required time. The supernatant was monitored periodically by gas chromatography (GC) analysis with 1,4-chlorobenzene as the internal standard.

### Cycloaddition reaction between epibromohydrin and CO_2_

A round-bottomed flask was charged with the catalyst (1 mg Co-TCPP, 20 mg ZIF-8, 56 mg Co-TCPP@ZIF-8 or 20 mg Co-TCPP@H-LDH@ZIF-8 containing 1 mg Co-TCPP), tetraethylammonium bromide (22 mg) and substrate (0.2 mmol) in acetonitrile (0.4 mL) and N,N-dimethylformamide (DMF) (0.1 mL). After stirring under 1 atm CO_2_ at room temperature for the required time, the reaction was monitored periodically by analyzing the sample with GC analysis. For reusability examination of Co-TCPP@H-LDH@ZIF-8, the heterogeneous mixture was centrifugated after the reaction and the recovered catalyst was then reused for the subsequent reaction with fresh substrate and solvent.

### CO_2_ cycloaddition reactions with different-sized epoxides

Typically, a round-bottomed flask (5 mL) was charged with the catalyst (1 mg Co-TCPP or 20 mg Co-TCPP@H-LDH@ZIF-8 containing 1 mg Co-TCPP), tetraethylammonium bromide (TEAB) and epoxides (epibromohydrin, styrene oxide or glycidyl phenyl ether) in DMF (0.1 mL) and acetonitrile (0.4 mL). The reaction mixture was then stirred at room temperature for the required time. The reaction was monitored by analyzing the sample with GC analysis.

### Cycloaddition reaction between CO_2_ and small-size epoxides with various functional groups

A round-bottomed flask was charged with the catalyst (20 mg Co-TCPP@H-LDH@ZIF-8), co-catalyst (22 mg TEAB or 64 mg tetrabutylammonium bromide) and epoxides (0.2 mmol) in acetonitrile (0.4 mL) and DMF (0.1 mL). After stirring under 1 atm CO_2_ at room temperature for the required time, the reaction was monitored by analyzing the sample with GC analysis.

### One-pot oxidation/Knoevenagel condensation of various alcohols

A round-bottomed flask was charged with the catalyst (0.5 mg of TAPP, 3.5 mg of H-LDH, 6 mg of MOF-74, 10 mg of TAPP@MOF-74 or 10 mg of TAPP@H-LDH@MOF-74), ethyl cyanoacetate (0.2 mmol), alcohol (0.04 mmol) and 2,2,6,6-tetramethylpiperidine 1-oxyl (3 mg) in toluene (1 mL). After stirring under 1 atm of O_2_ at 80°C for the required time, the reaction was monitored periodically by GC analysis. For reusability investigation of TAPP@H-LDH@MOF-74, the heterogeneous mixture was centrifugated after the reaction and the recovered catalyst was then reused for the subsequent reaction with fresh substrate and solvent.

## Supplementary Material

nwz147_Supplemental_File
