# Supplementary material for: Encapsulating soluble active species into hollow crystalline porous capsules beyond integration of homogeneous and heterogeneous catalysis
Source: Natl Sci Rev. 2019 Oct 1;7(1):37–45. doi: 10.1093/nsr/nwz147 (PMC8288971; doi:10.1093/nsr/nwz147)
Supplement: nwz147_Supplemental_File [file nwz147_supplemental_file.doc]

**Supplementary Information**

**Encapsulating Soluble Active Species into Hollow Crystalline Porous Capsules beyond Integration of Homogeneous and Heterogeneous Catalysis**

Guorui Cai, Meili Ding, Qianye Wu, and Hai-Long Jiang*

Hefei National Laboratory for Physical Sciences at the Microscale, CAS Key Laboratory of Soft Matter Chemistry, Collaborative Innovation Center of Suzhou Nano Science and Technology, Department of Chemistry, University of Science and Technology of China, Hefei, Anhui 230026, P.R. China.

*Correspondence and requests for materials should be addressed to H.-L.J (email: [jianglab@ustc.edu.cn](mailto:jianglab@ustc.edu.cn)).

**Supplementary Materials and Instrumentation.**

All chemicals were purchased from commercial sources and used without any further purification: cobalt nitrate hexahydrate (99%, Energy Chemical), zinc nitrate hexahydrate (99%, Energy Chemical), nickel nitrate hexahydrate (98%, Energy Chemical), 2-methylimidazole (98%, Shanghai Macklin Biochemical Reagent Co., Ltd.), 2,5-dihydroxyterephthalic acid (98%, Energy Chemical), methanol (AR, Sinopharm Chemical Reagent Co., Ltd.), ethanol (AR, Sinopharm Chemical Reagent Co., Ltd.), N,N-dimethylformamide (AR, Sinopharm Chemical Reagent Co., Ltd.), acetonitrile (AR, Sinopharm Chemical Reagent Co., Ltd.), ethyl acetate (AR, Sinopharm Chemical Reagent Co., Ltd.), hydrochloric acid (AR, Sinopharm Chemical Reagent Co., Ltd.), nitric acid (AR, Sinopharm Chemical Reagent Co., Ltd.), coomassie brilliant blue R_250_ (AR, Sinopharm Chemical Reagent Co., Ltd.), *n*-octylamine (99%, Aladdin Industrial Inc.), tetraethylammonium bromide (TEAB, AR, Sinopharm Chemical Reagent Co., Ltd.), tetrabutylammonium bromide (TBAB, AR, Sinopharm Chemical Reagent Co., Ltd.), epibromohydrin (97%, Energy Chemical), epichlorohydrin (AR, Sinopharm Chemical Reagent Co., Ltd.), glycidol (97%, Energy Chemical), 1, 2-epoxyhexane (97%, Energy Chemical), styrene oxide (98%, Energy Chemical), glycidyl phenyl ether (98%, Energy Chemical), toluene (AR, Sinopharm Chemical Reagent Co., Ltd.), benzyl alcohol (AR, Sinopharm Chemical Reagent Co., Ltd.), 4-chlorobenzyl alcohol (98%, Energy Chemical), 4-bromobenzyl alcohol (98%, Energy Chemical), 4-nitrobenzyl alcohol (99.5%, Energy Chemical), 4-methylbenzyl alcohol (99%, Energy Chemical), 4-methoxybenzyl alcohol (99%, Energy Chemical), ethyl cyanoacetate (99%, Aladdin Industrial Inc.), 2,2,6,6- tetramethylpiperidine 1-oxyl (98.5%, Energy Chemical).

Deionized water was obtained by reversed osmosis with the specific resistance of 18.25 MΩ∙cm followed by ion-exchange and filtration (Cleaned Water Treatment Co., Ltd., Hefei). Powder X-ray diffraction (XRD) patterns were conducted on a Holland X’Pert PRO fixed anode X-ray diffractometer or Japan Rigaku SmartLabTM rotation anode X-ray diffractometer equipped with graphite monochromatized Cu Kα radiation (λ = 1.54 Å). Field-emission scanning electron microscopy (SEM) was performed with a field emission scanning electron microanalyzer (Zeiss Supra 40 scanning electron microscope) operating at an acceleration voltage of 5 kV. The transmission electron microscopy (TEM), high-angle annular dark-field scanning transmission electron microscopy (HAADF-STEM) and energy-dispersive X-ray spectroscopy (EDS) line scanning were carried out on JEOL-2010, JEOL ARM-200F and JEOL-2100F with an electron acceleration energy of 200 kV. The N_2_ and CO_2_ sorption isotherms were acquired by using the automatic volumetric adsorption equipment (Micromeritics ASAP 2020). The contents of Co and Mn-centered porphyrin in the composites were quantified by an inductively coupled plasma atomic emission spectrometer (ICP-AES, Optima 7300 DV). UV-Vis spectra of the samples were collected by using a spectrophotometer (Shimadzu UV-2700). The conversion and yields of catalytic reaction products were identified and analyzed on a Shimadzu gas chromatograph (GC-2010 Plus with a 0.25 mm × 30 m Rtx®-5 capillary column).

**Synthesis of H-LDH**

H-LDH was synthesized according to the documented method with some modifications [S1]. Typically, a Co-MOF (ZIF-67) template was prepared firstly through mixing of cobalt nitrate hexahydrate (747 mg) and 2-methylimidazole (984 mg) in methanol solution (150 mL) at room temperature for 24 h. The precipitate was isolated by centrifugation and washed with ethanol for several times. The obtained template material was dispersed in ethanol (25 mL) for further use.

The as-prepared mixture was added into an ethanol solution (6 mg/mL, 50 mL) of cobalt nitrate hexahydrate and then stirred at 90 ^o^C for 2 h. Upon cooling down, the product was collected by centrifugation and washed with ethanol for several times. The obtained sample was then aged in ethanol (20 mL) for several days.

**Synthesis of H-LDH@ZIF-67**

Typically, 2-methylimidazole (1.64 g) was dissolved in a mixture of ethanol (3 mL) and deionized water (5 mL). Then the ethanol solution of H-LDH (2 mL) was added. The reaction was allowed to proceed at room temperature for 5 h. The precipitate was recovered by centrifugation, washed with methanol for several times, and then dried in a vacuum oven at 85 ^o^C for 12 h to give H-LDH@ZIF-67.

**Synthesis of H-LDH@MOF-74**

In a typical experiment, the H-LDH solution (2 mL) was centrifuged, washed with N,N-dimethylformamide (DMF) for several times, and then dispersed in DMF (1 mL). Then a DMF solution (4 mg/mL, 1 mL) of 2,5-dihydroxyterephthalic acid was added. After stirring for several minutes at room temperature, a DMF solution (12 mg/mL, 1 mL) of nickel nitrate hexahydrate was added. The mixture was then sealed up in a glass vial (5 mL) and heated at 120 ^o^C for 2 h. The resultant sample was recovered by centrifugation, washed with methanol for several times, and then dried in a vacuum oven at 85 ^o^C for 12 h to give H-LDH@MOF-74.

**The *in-situ* encapsulation of metalloporphyrins**

The Co-TCPP (5, 10, 15, 20-tetrakis(4-carboxyphenyl)porphyrin cobalt, Supplementary Scheme 1) was obtained according to the previous report [S2]. The synthetic procedure for Co-TCPP@H-LDH@ZIF-8 was as follows. Typically, 8 mL of ethanol solution of H-LDH was isolated by centrifugation and washed with methanol for several times. The obtained sample dispersed in methanol (1mL) was soaked in methanol solution (8 mg/mL, 1 mL) of Co-TCPP. After stirring for 1 h at room temperature, a methanol solution (45 mg/mL, 2 mL) of 2-methylimidazole was added under gentle stirring, and then methanol solution (10 mg/mL, 2 mL) of zinc nitrate hexahydrate was added. After proceeding at room temperature for 5 h, the precipitate was washed with methanol several times and then dried in a vacuum oven at 85 ^o^C for 12 h to give Co-TCPP@H-LDH@ZIF-8.

The synthetic procedure for Co-TCPP@ZIF-8 was followed by the preparation of Co-TCPP@H-LDH@ZIF-8, except for that the methanol solution (60 mL) of Co-TCPP (20 mg), 2-methylimidazole (1.8 g) and zinc nitrate hexahydrate (400 mg) was stirred at room temperature for 24 h.

**Supplementary Scheme 1. The molecular structure of Co-TCPP.**


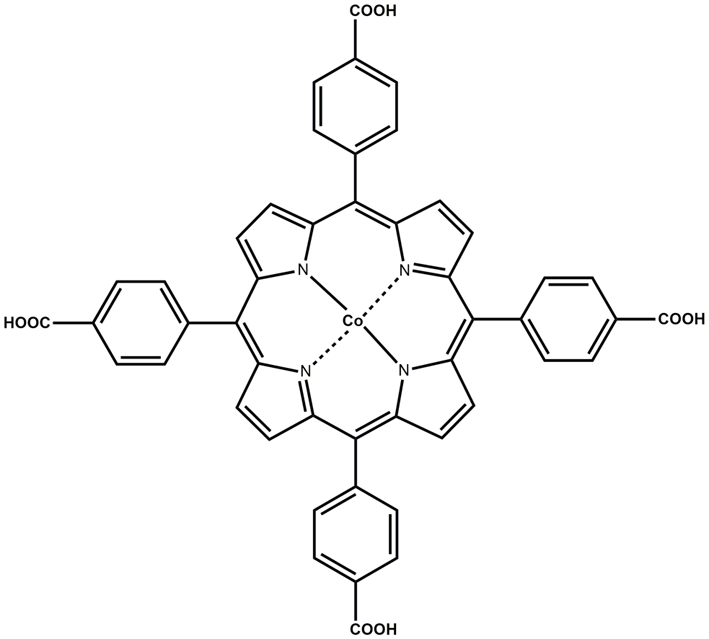


The Mn-TCPPNa obtained through stirring NaOH with Mn-TCPP ([5, 10, 15, 20-tetra(4-carboxyphenyl)porphyrin] manganese, Supplementary Scheme 2) [S2]. The synthetic procedure for Mn-TCPPNa@H-LDH@ZIF-8 was followed by the preparation of Co-TCPP@H-LDH@ZIF-8, except for that the Co-TCPP was replaced by the Mn-TCPPNa (8 mg).

The synthetic procedure for Mn-TCPPNa@ZIF-8 was followed by the preparation of Co-TCPP@ZIF-8, except for that the Co-TCPP was replaced by the Mn-TCPPNa (20 mg).

**Supplementary Scheme 2. The molecular structure of Mn-TCPPNa.**


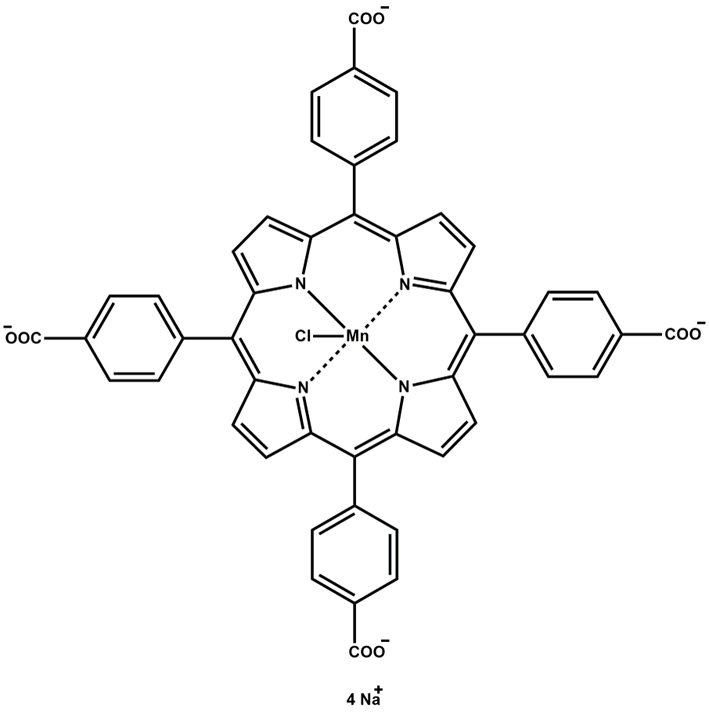


The TAPP (5, 10, 15, 20-tetrakis(4-aminophenyl)porphyrin, Supplementary Scheme 3) was obtained according to the previous report [S3]. The synthetic procedure for TAPP@H-LDH@MOF-74 was as follows: In a typical experiment, 8 mL of ethanol solution of H-LDH was isolated by centrifugation and washed with DMF for three times. The obtained sample was soaked in DMF solution (2 mg/mL, 4 mL) of TAPP. After stirring for 1 h at room temperature, a DMF solution (4 mg/mL, 4 mL) of 2,5-dihydroxyterephthalic acid was added under gentle stirring, and then DMF solution (15 mg/mL, 4 mL) of copper nitrate hydrate was added. After proceeding at 120 ^o^C for 2 h, the precipitate was washed with methanol for several times and then dried in a vacuum oven at 85 ^o^C for 12 h to give TAPP@H-LDH@MOF-74.

The synthetic procedure for TAPP@MOF-74 was as follows: TAPP (8 mg), copper nitrate hydrate (60 mg) and 2,5-dihydroxyterephthalic acid (16 mg) were stirred in DMF (12 mL) solution for several minutes at room temperature. The mixture was then sealed up in a glass vial (20 mL) and heated at 120 ^o^C for 5 h. The resultant sample was recovered by centrifugation, washed with DMF and methanol for several times, and then dried in a vacuum oven at 85 ^o^C for 12 h to give TAPP@MOF-74.

**Supplementary Scheme 3. The molecular structure of TAPP.**


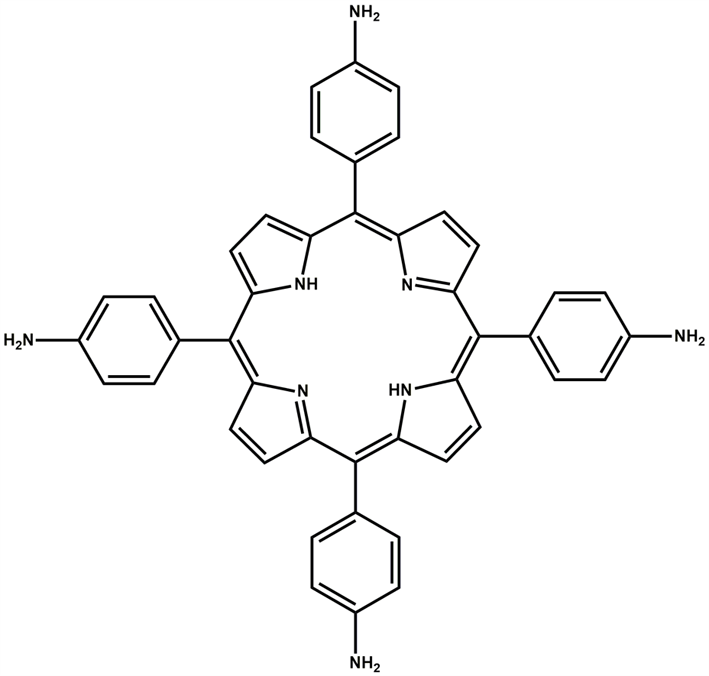


**The solution-phase epibromohydrin adsorption studies**

Typically, 20 mg of Co-TCPP@H-LDH@ZIF-8 was soaked in acetonitrile solution of epibromohydrin (1 mL, 1 mol/L) and stirred at room temperature. The concentration of epibromohydrin (C_i_) in the supernatant was analyzed and monitored periodically by GC with 1,4-chlorobenzene as the internal standard. The adsorption capacity of epibromohydrin was calculated according to the following equation：

𝑞_𝑡_ = (𝐶_𝑖_ ‒ 𝐶_𝑓_)**·**𝑉**·**M/m

q_t_: the amount of adsorbate adsorbed at time t (g/g catalyst);

C_i_: the initial concentration of the adsorbate (mol/L);

C_f_: the final concentration of the adsorbate after adsorption (mol/L);

V: the volume of solution (L);

M: the molar mass of adsorbate (g/mol);

m: the amount of adsorbent added (g).


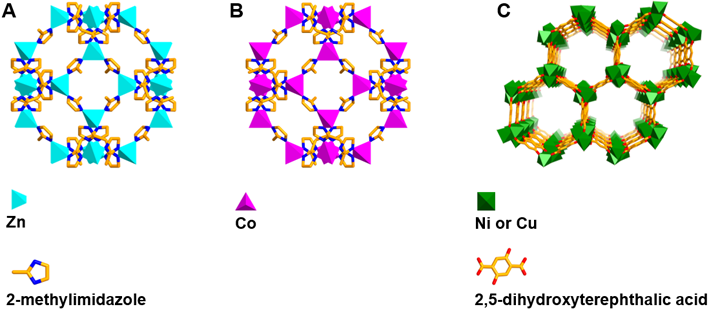


**Supplementary Figure 1.** **View of the crystal structures.** Crystal structures of (**A**) ZIF-8 (Zn(2-methylimidazole)_2_), (**B**) ZIF-67 (Co(2-methylimidazole)_2_), (**C**) MOF-74 (Ni_2_(2,5-dihydroxyterephthalic acid) or Cu_2_(2,5-dihydroxyterephthalic acid).


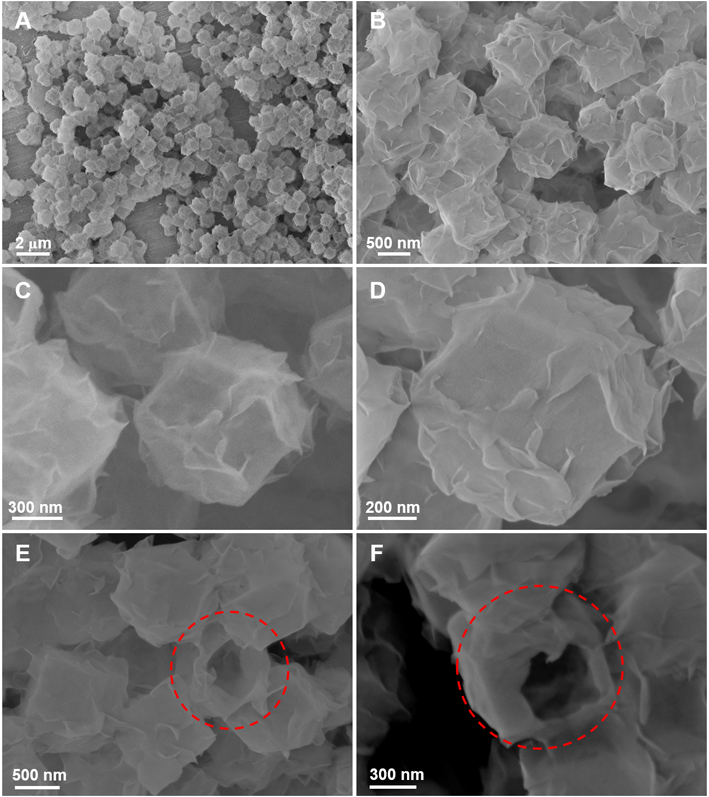


**Supplementary Figure 2.** **SEM images.** Representative SEM images for H-LDH at different magnifications. The broken capsules highlighted in red circles reveal the presence of a hollow interior space in the H-LDH template.


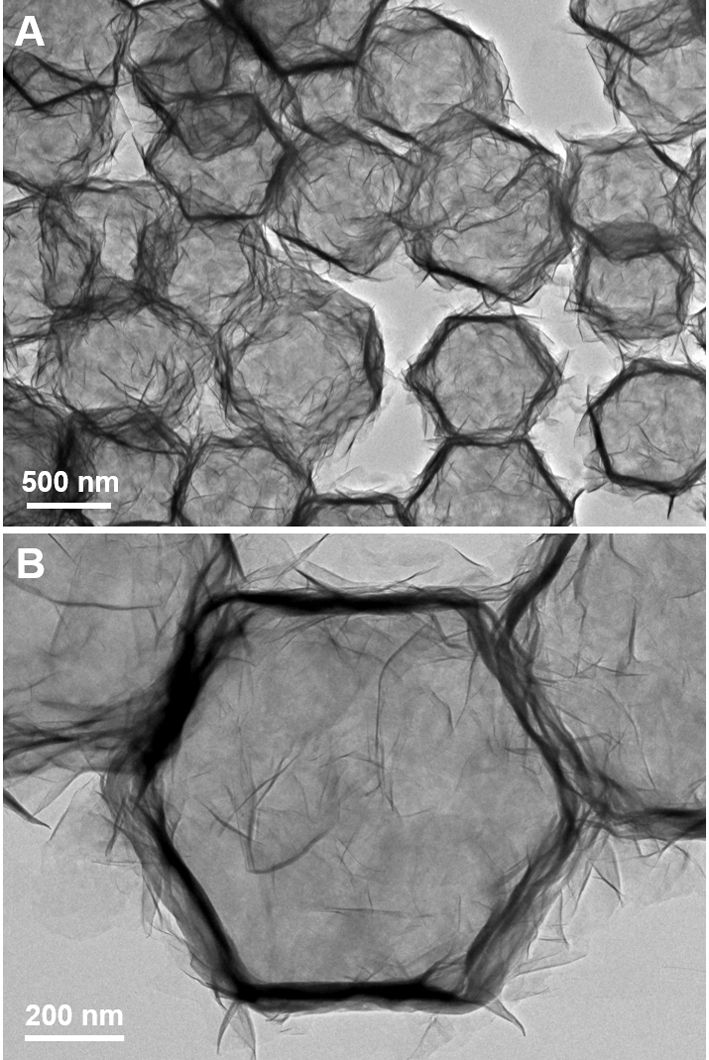


**Supplementary Figure 3.** **TEM images.** Representative (**A**) low-magnification and (**B**) high-magnification TEM images for H-LDH.


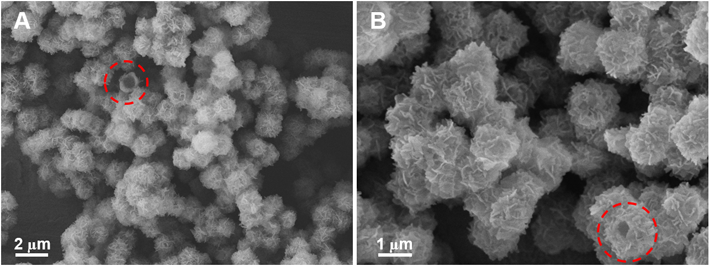


**Supplementary Figure 4. SEM images.** Representative SEM images for H-LDH@ZIF-8 at different magnifications. The broken capsules highlighted in red circles reveal the presence of a hollow interior space in the H-LDH@ZIF-8.


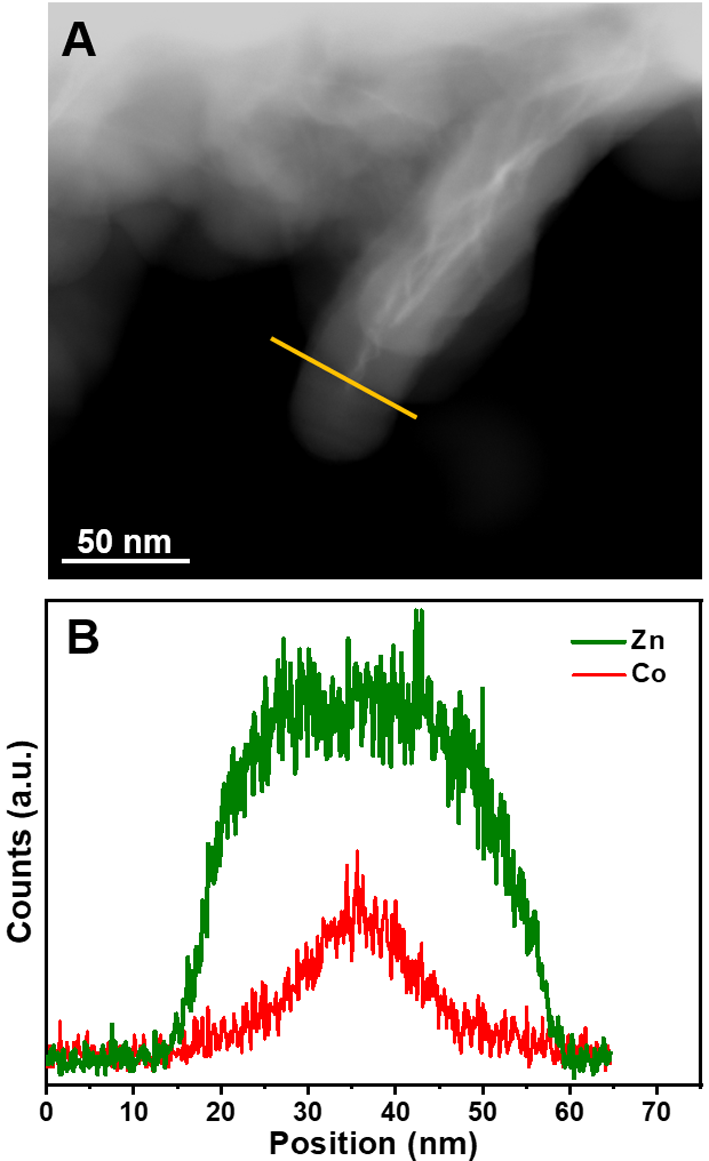


**Supplementary Figure 5. The HAADF-STEM image and EDS line scanning profiles.** (**A**) The HAADF-STEM images and (**B**) EDS line scanning profiles for the nanoflake of H-LDH@ZIF-8, clearly showing the core-shell structure.


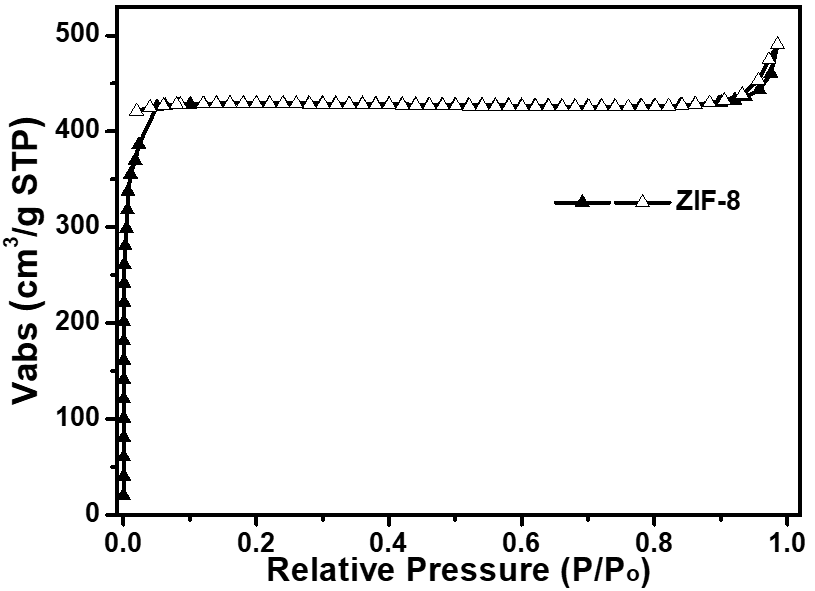


**Supplementary Figure 6. The N_2_ sorption isotherms of ZIF-8 at 77 K.**

**
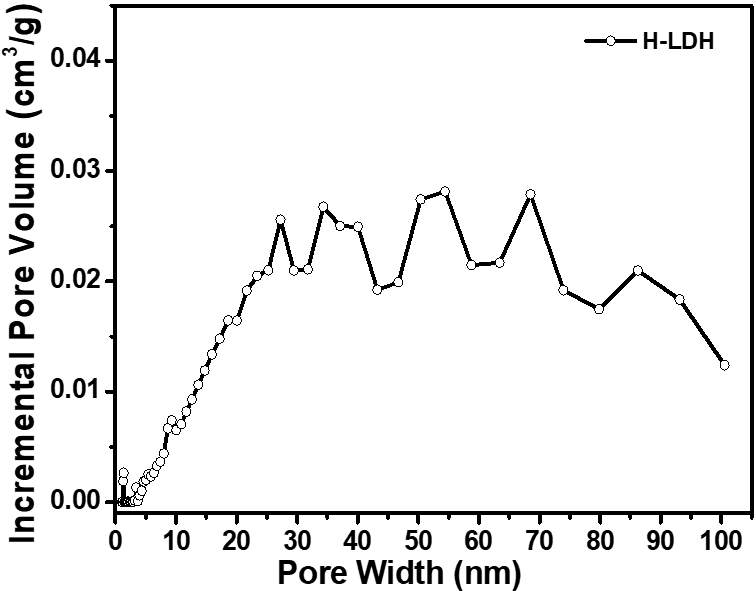
**

**Supplementary Figure 7. The pore size distribution (DFT method) of H-LDH template.**


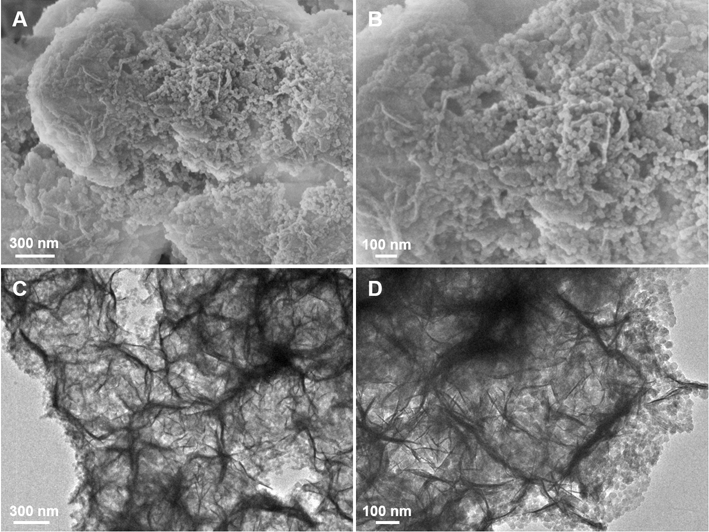


**Supplementary Figure 8. SEM and TEM images.** (**A** and **B**) SEM and (**C** and **D**) TEM images of the product obtained by the attempted growth of H-LDH@ZIF-8 when the metal salts were added prior to ligands.

When the metal salts were added prior to ligands, Zn^2+^ tended to be repelled from the LDH surface due to its positively charged skeleton. Upon the subsequent addition of ligand solution, the MOF was prone to nucleate independently in the solution rather than on the template. The above corresponding SEM and TEM images (Supplementary Fig. 4) clearly demonstrate that only a few ZIF-8 nanocrystals were partially located on the H-LDH. On the contrary, the ligand solution added in advance would be preferentially adsorbed onto H-LDH templates to act as growth sites, facilitating the heterogeneous nucleation of ZIF-8. The results are well supported by SEM and TEM observation, showing that a continuous ZIF-8 layer was uniformly anchored onto the surface of H-LDH (Fig. 2A and B). These two routes reveal that the adding sequence for the metal and ligand precursors plays a critical role in the growth of MOF shell onto the H-LDH. The strong interaction between the ligands and the coordinatively unsaturated metal sites on the template is believed to be an important driving force for the directed growth. In addition, for the heterogeneous nucleation of ZIF-8, the deprotonation of the imidazole ligand is an essential step, which is promoted by the H-LDH template owing to its abundant basic groups (i.e., OH^-^) [S4, S5].


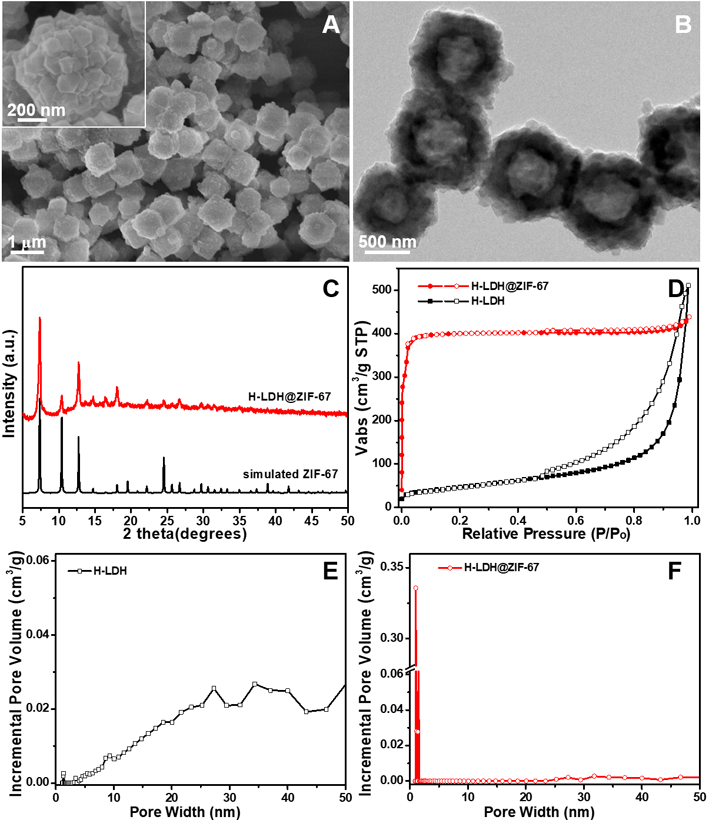


**Supplementary Figure 9. SEM and TEM images, powder XRD patterns, and N_2_ sorption isotherms.** (**A**) SEM and (**B**) TEM images of H-LDH@ZIF-67. Insert: the enlarged SEM image. (**C**) Powder XRD patterns of H-LDH@ZIF-67 and simulated ZIF-67. (**D**) N_2_ sorption isotherms of H-LDH@ZIF-67 at 77 K. The pore size distribution (DFT method) of (**E**) H-LDH template and (**F**) H-LDH@ZIF-67.


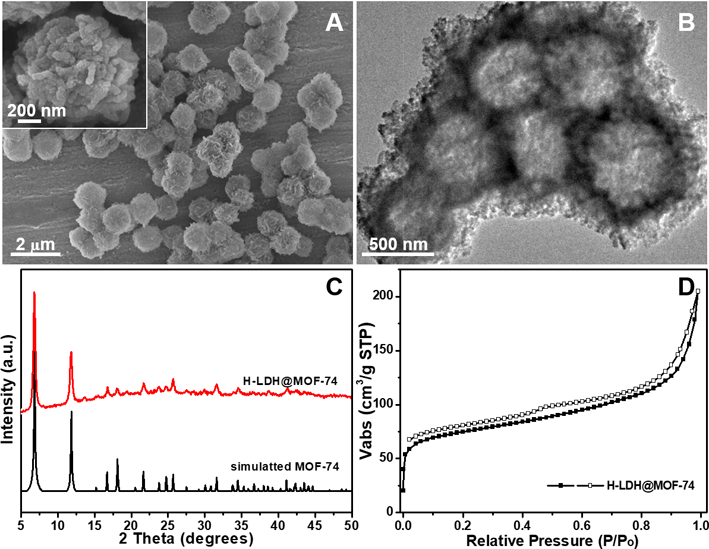


**Supplementary Figure 10.** **SEM and TEM images, powder XRD patterns, and N_2_ sorption isotherms.** The (**A**) SEM and (**B**) TEM images of H-LDH@MOF-74. Insert: the enlarged SEM image. (**C**) Powder XRD patterns of H-LDH@MOF-74 and simulated MOF-74. (**D**) N_2_ sorption isotherms of H-LDH@MOF-74 at 77 K.


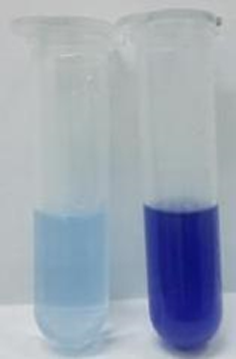


**Supplementary Figure 11.** **Photographs.** Photographs of the methanol solution of (left) R_250_@ZIF-8 and (right) R_250_@H-LDH@ZIF-8 after acid etching, indicating that much more R_250_ are loaded into the latter.


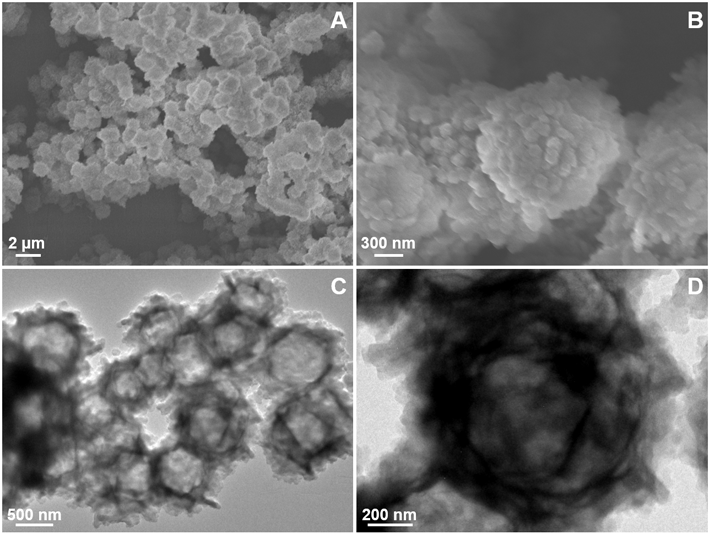


**Supplementary Figure 12. SEM and TEM images.** The (**A** and **B**) SEM and (**C** and **D**) TEM images of Co-TCPP@H-LDH@ZIF-8.


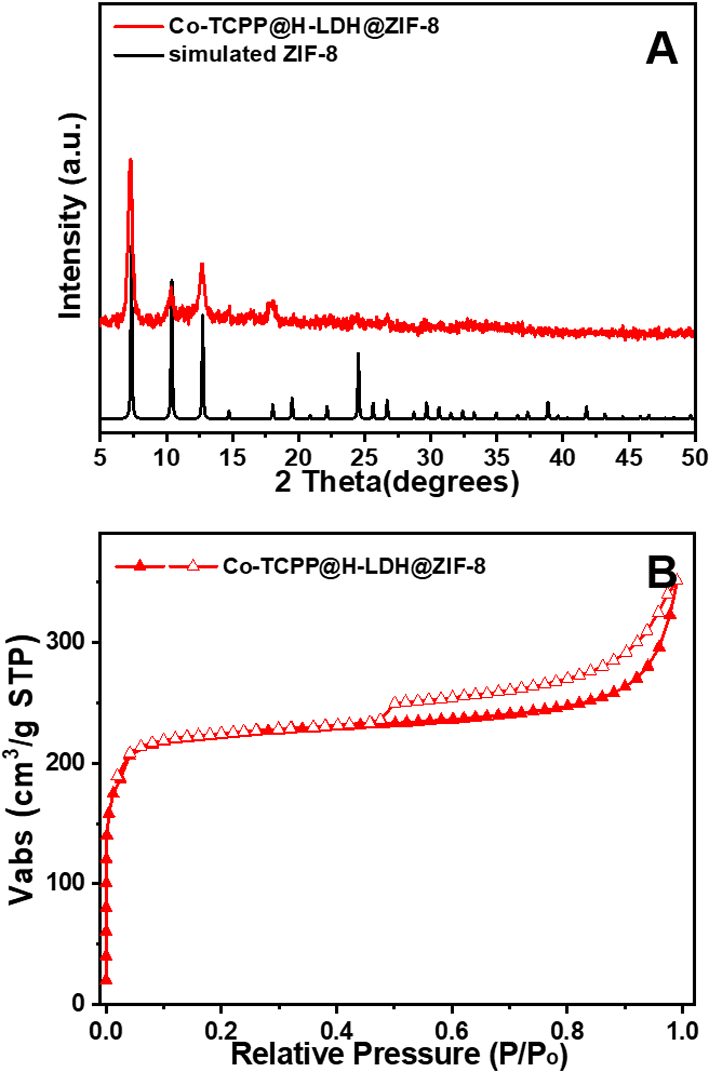


**Supplementary Figure 13. Powder XRD patterns and N_2_ sorption isotherms.** (**A**) Powder XRD patterns of Co-TCPP@H-LDH@ZIF-8 and simulated ZIF-8. (**B**) N_2_ sorption isotherms of Co-TCPP@H-LDH@ZIF-8 at 77 K.


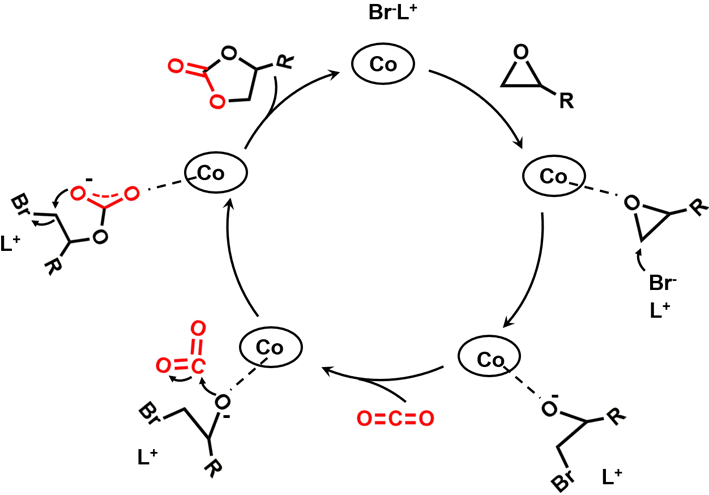


**Supplementary Figure 14.** **The proposed mechanism.** The proposed mechanism for CO_2_ cycloaddition with epoxides over Co-TCPP@H-LDH@ZIF-8 involving Co-TCPP active species, in the presence of TEAB (Br^-^L^+^).


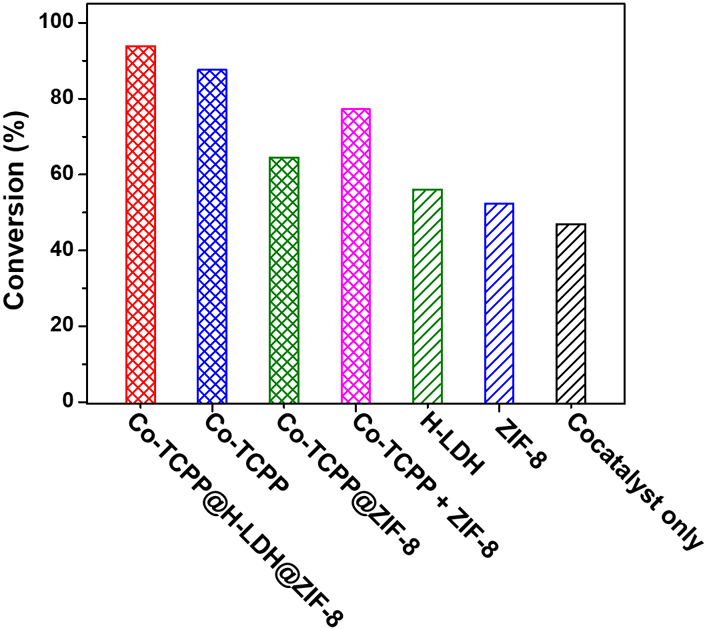


**Supplementary Figure 15.** **The cycloaddition reaction conversions between CO_2_ and epibromohydrin.** Comparison of the cycloaddition reaction conversions between CO_2_ and epibromohydrin over different catalysts in the presence of TEAB as cocatalyst. Conditions: substrate (0.2 mmol), Co-TCPP@H-LDH@ZIF-8 (20 mg), acetonitrile (0.4 mL), DMF (0.1 mL), TEAB (22 mg), under 1 atm of CO_2_, room temperature, 2 d.

The above results indicate that the hollow MOF composites confer superior activity to the bulk MOF counterpart, active homogeneous guest, their physical mixture and also solid composite. Though the free Co-TCPP and its corresponding physical mixture (Co-TCPP + ZIF-8) seem to afford comparable catalytic activity toward CO_2_ cycloaddition reaction, unfortunately, they are hard to be separated/recyclable due to the homogeneous nature in the reaction solution.

The control experiment over ZIF-8 or H-LDH template gives a similar conversion to the blank experiment with TEAB cocatalyst only, indicating that the MOF shells in the Co-TCPP@H-LDH@ZIF-8 do not act as active species, while the Co-TCPP yolks are the main active components, as also confirmed by the size-selective catalysis (Fig. 5C).


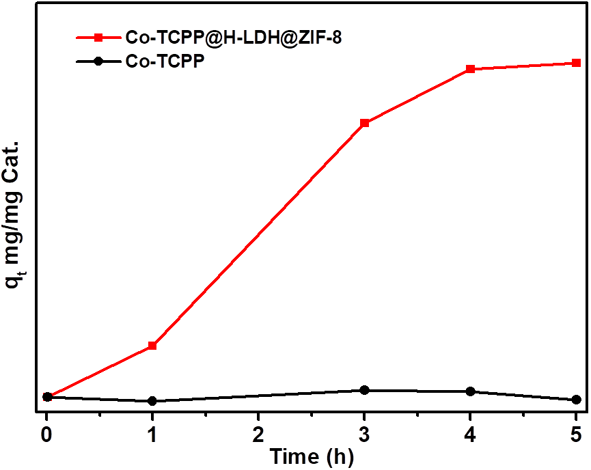


**Supplementary Figure 16.** **The adsorption of epibromohydrin.** Adsorption of epibromohydrin from acetonitrile along with time by Co-TCPP and Co-TCPP@H-LDH@ZIF-8.

The liquid-phase adsorption experiment results indicate that the Co-TCPP@H-LDH@ZIF-8 displays a more effective epibromohydrin adsorption rate and amount than the Co-TCPP complex, clearly supporting the more favorable enrichment effect for the substrate in the former. It is expected that the local concentration increase of the liquid-phase substrate around the active sites would be favorable to accelerate the cycloaddition reaction [S6, S7].


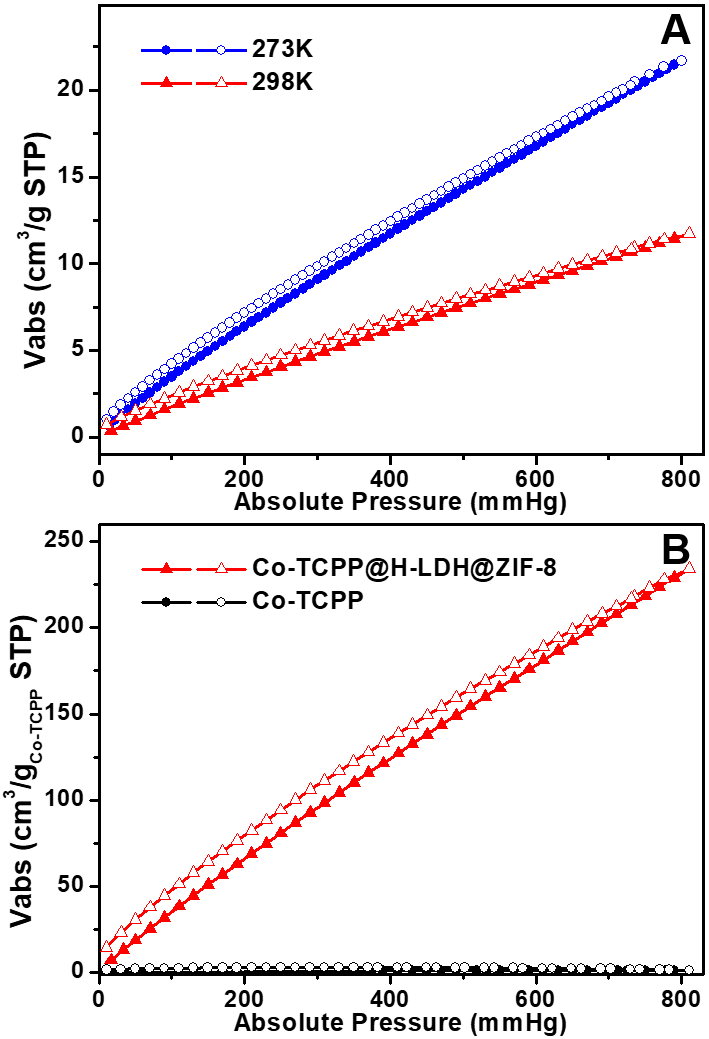


**Supplementary Figure 17.** **CO_2_ sorption isotherms.** (**A**) CO_2_ sorption isotherms of Co-TCPP@H-LDH@ZIF-8 at 273 K and 298 K. (**B**) CO_2_ sorption isotherms of free Co-TCPP and H-LDH@ZIF-8 encapsulated Co-TCPP at 298 K. The unit of y-axis in (**B)** is calculated based on the quality of Co-TCPP, to highlight the difference in CO_2_ adsorption between the samples of Co-TCPP in the presence or absence of MOFs as the shells.

The results indicate that the Co-TCPP@H-LDH@ZIF-8 display a significantly increased CO_2_ adsorption amount compared to the free Co-TCPP, clearly supporting the more favorable enrichment for the gas-phase substrate in the former. It is expected that the increase of local CO_2_ concentration around the active sites would boost the CO_2_ cycloaddition reaction [S8].


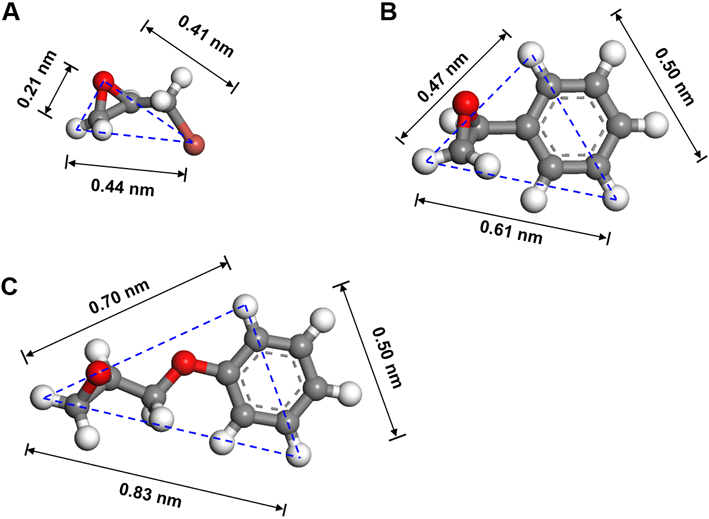


**Supplementary Figure 18.** **View of substrate molecular sizes.** Schematic showing the molecular sizes of (**A**) epibromohydrin, (**B**) styrene oxide, (**C**) glycidyl phenyl ether. These data were measured by materials studio.

The molecular size of epibromohydrin is smaller than the opening size of ZIF-8 (window size: 0.34 nm), so it is easy to penetrate into the MOF capsule. In contrast, styrene and glycidyl phenyl ether have bigger sizes than the window size of ZIF-8，causing the steric hindrance. Therefore, the Co-TCPP encapsulated in hollow MOF capsule would be able to show size-selective catalysis.


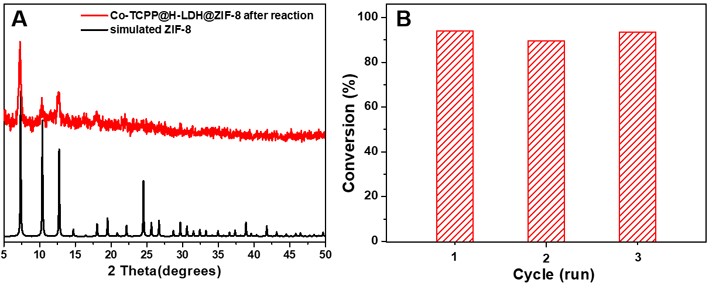


**Supplementary Figure 19.** **Recycling tests.** (**A**) XRD pattern of Co-TCPP@H-LDH@ZIF-8 after reaction. (**B**) Recycling tests in the CO_2_ cycloaddition with epibromohydrin over Co-TCPP@H-LDH@ZIF-8. Reaction conditons: 0.2 mmol substrate, 20 mg catalyst, 0.4 mL acetonitrile, 0.1 mL DMF, 22 mg TEAB, 1 atm CO_2_, at room temperature, 2 d.

**Supplementary Table 1.** Cycloaddition reaction between CO_2_ and epoxides with different functional groups catalyzed by Co-TCPP@H-LDH@ZIF-8.^a^


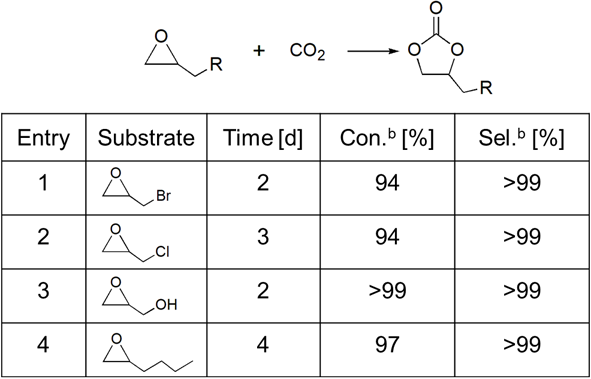


^a^Reaction conditions: substrate (0.2 mmol), catalyst (20 mg), acetonitrile (0.4 mL), DMF (0.1 mL), co-catalyst (22 mg of TEAB for entry 1, 2; 64 mg of TBAB for entry 3, 4), 1 atm CO_2_, room temperature. ^b^Determined by GC.


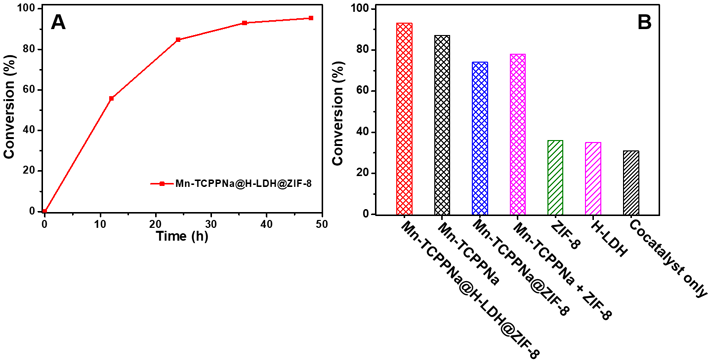


**Supplementary Figure 20.** **The cycloaddition reaction conversion between CO_2_ and epibromohydrin.** (**A**) Time conversion of the cycloaddition reactions between CO_2_ and epibromohydrin over Mn-TCPPNa@H-LDH@ZIF-8. (**B**) Comparison of the cycloaddition reaction conversion between CO_2_ and epibromohydrin over different catalysts in the presence of TEAB cocatalyst. Reaction conditions: 0.2 mmol substrate, 20 mg Mn-TCPPNa@H-LDH@ZIF-8, 0.4 mL acetonitrile, 0.1 mL DMF, 22 mg TEAB, 1 atm CO_2_, room temperature, 36h.

The results indicate that the hollow MOF composites confer superior activity to the bulk MOF counterpart, active homogeneous guest, their physical mixture and solid composite. Though the free Mn-TCPPNa and its corresponding physical mixtures (Mn-TCPPNa + ZIF-8) seem to afford comparable activity toward CO_2_ cycloaddition reaction, unfortunately, they are hard to be separated/recyclable due to the homogeneous nature in the reaction solution.


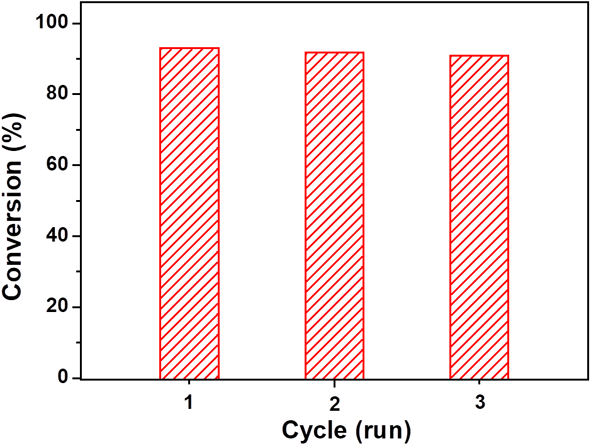


**Supplementary Figure 21.** **Recycling tests.** Recycling tests in the CO_2_ cycloaddition with epibromohydrin over Mn-TCPPNa@H-LDH@ZIF-8. Reaction conditions: substrate (0.2 mmol), catalyst (20 mg), acetonitrile (0.4 mL), DMF (0.1 mL), TEAB (22 mg), CO_2_ (1 atm), room temperature, 36 h.


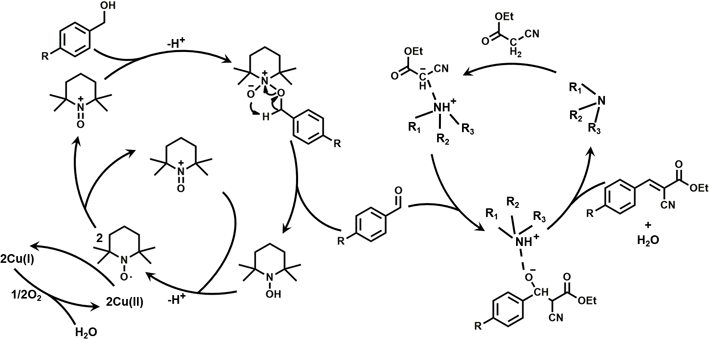


**Supplementary Figure 22.** **The proposed reaction mechanism.** Proposed reaction mechanism for the one-pot oxidation/Knoevenagel condensation reaction over TAPP@H-LDH@MOF-74 [S9, S10]: the oxidation reaction of alcohols is catalyzed by Cu-MOF-74 shell in the presence of 2,2,6,6-tetramethylpiperidine 1-oxyl; the subsequent Knoevenagel condensation reaction is catalyzed by the TAPP yolk.


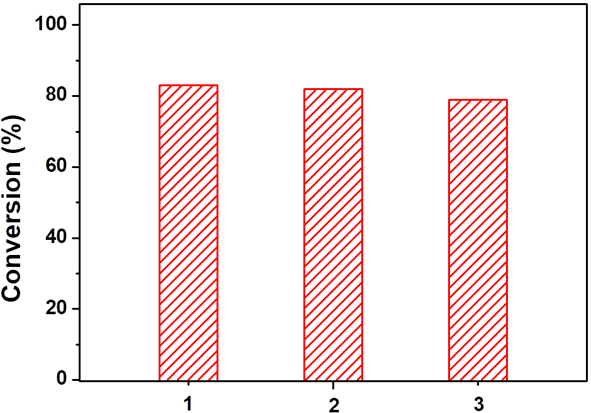


**Supplementary Figure 23.** **Recycling tests.** Recycling tests in the one-pot oxidation/Knoevenagel condensation reaction over TAPP@H-LDH@MOF-74. Reaction conditons: benzyl alcohol (0.04 mmol), ethyl cyanoacetate (0.2 mmol), 2,2,6,6- tetramethylpiperidine 1-oxyl (3 mg), catalyst (10 mg), toluene (1 mL), 1 atm O_2_, 80 ^o^C, 5 h.

**Supplementary Table 2.** One-pot oxidation/Knoevenagel condensation of alcohols with different functional group over TAPP@H-LDH@MOF-74^a^


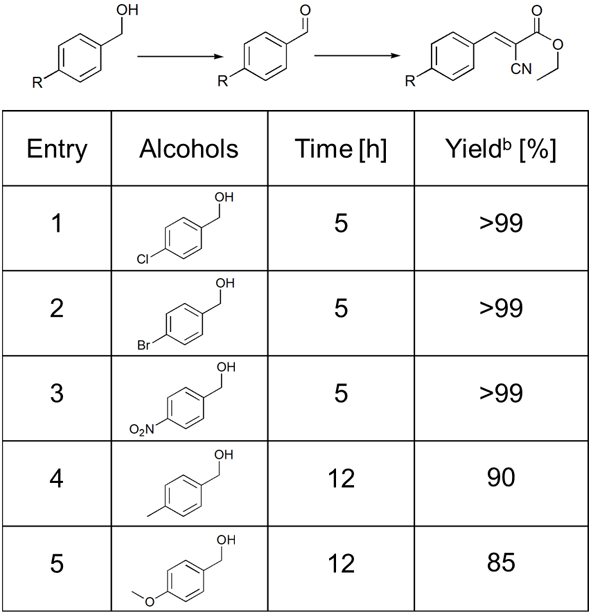


^a^Reaction conditions: alcohol (0.04 mmol), ethyl cyanoacetate (0.2 mmol), 2,2,6,6- tetramethylpiperidine 1-oxyl (3 mg), catalyst (10 mg), toluene (1 mL), 1 atm O_2_, 80 ^o^C. ^b^Determined by GC.

**Supplementary References**

S1. Jiang Z, Li Z and Qin Z *et al.* LDH nanocages synthesized with MOF templates and their high performance as supercapacitors. *Nanoscale* 2013; **5**: 11770-5.

S2. Feng D, Gu ZY and Li JR *et al.* Zirconium-metalloporphyrin PCN-222: mesoporous metal-organic frameworks with ultrahigh stability as biomimetic catalysts. *A**ngew Chem Int Ed* 2012; **51**: 10307-10.

S3. Yuasa M, Oyaizu K and Yamaguchi A *et al.* Micellar cobaltporphyrin nanorods in alcohols. *J Am Chem Soc* 2004; **126**: 11128-9.

S4. Liu Y, Wang N and Pan JH *et al.* In situ synthesis of MOF membranes on ZnAl-CO_3_ LDH buffer layer-modified substrates. *J Am Chem Soc* 2014; **136**: 14353-6.

S5. Zhao J, Nunn WT and Lemaire PC *et al.* Facile conversion of hydroxy double salts to metal-organic frameworks using metal oxide particles and atomic layer deposition thin-film templates. *J Am Chem Soc* 2015; **137**: 13756-9.

S6. Xiao DJ, Oktawiec J and Milner PJ *et al.* Pore environment effects on catalytic cyclohexane oxidation in expanded Fe_2_(dobdc) analogues. *J Am Chem Soc* 2016; **138**: 14371-9.

S7. Liu F, Wang L and Sun Q *et al.* Transesterification catalyzed by ionic liquids on superhydrophobic mesoporous polymers: heterogeneous catalysts that are faster than homogeneous catalysts. *J Am Chem Soc* 2012; **134**: 16948-50.

S8. Kajiwara T, Fujii M and Tsujimoto M *et al.* Photochemical reduction of low concentrations of CO_2_ in a porous coordination polymer with a ruthenium(II)-CO complex. *Angew Chem Int Ed* 2016; **55**: 2697-700.

S9. Hossaina MM and Shyu SG, Efficient and Selective Aerobic Alcohol Oxidation Catalyzed by Copper(II)/2,2,6,6,-Tetramethylpiperidine-1-oxyl at Room Temperature. *Adv Synth Catal* 2010; **352**: 3061-3068.

S10. Tan YC and Zeng HC, Lewis basicity generated by localised charge imbalance in noble metal nanoparticle-embedded defective metal–organic frameworks. *Nat Comm* 2018; **9**: 4326.
